# Antenatal care dropout and associated factors among mothers delivering in public health facilities of Dire Dawa Town, Eastern Ethiopia

**DOI:** 10.1186/s12884-021-04107-7

**Published:** 2021-09-15

**Authors:** Dereje Worku, Daniel Teshome, Chalachew Tiruneh, Alemtsehay Teshome, Gete Berihun, Leykun Berhanu, Zebader Walle

**Affiliations:** 1 Department of Gynecology and Obstetrics, College of Medicine and Health Sciences, Wachamo University, Hossana, Ethiopia; 2 Department of Anatomy, College of Medicine and Health Sciences, Wollo University, Dessie, Ethiopia; 3 Department of Environmental Health, College of Medicine and Health Sciences, Wollo University, Dessie, Ethiopia; 4 Department of Public Health, College of Health Sciences, Debre Tabor University, Debre Tabor, Ethiopia

**Keywords:** ANC, Dropout, Associated factor

## Abstract

**Introduction:**

More than two-thirds of the pregnant women in Africa have at least one antenatal care contact with a health care provider. However, to achieve the full life-saving potential that antenatal care promises for women and babies, four visits providing essential evidence-based interventions – a package often called focused antenatal care are required. Hence, identifying the factors associated with dropout of maternal health care utilization would have meaningful implications. The study aimed to assess antenatal care dropout and associated factors among mothers delivering in the public health facilities of Dire Dawa town, Ethiopia.

**Methods:**

Facility-based cross-sectional study was conducted from January 1 to 30, 2020. Proportionate sampling and simple random sampling techniques were used to select 230 women. Data were collected using a structured and pretested interview administered questionnaire during delivery. The data were entered into Epidata version 3.1 and analyzed using SPSS version 20. A binary logistic regression model with a 95 % confidence interval was used to analyze the results. Bivariable analysis (COR [crude odds ratio]) and multivariable analysis (AOR [adjusted odds ratio]) was used to analyze the results. From the bivariable analysis, variables with a *p-*value *<* 0.25 were entered into the multivariable logistic regression analysis. From the multivariable logistic regression analysis, variables with a significance level of *p*-value *<* 0.05 were taken as factors independently associated with ANC dropout.

**Result:**

The proportion of antenatal care dropouts was 86 (37.4 %) (95 % CI: 31.3–43.9). In logistic regression analyses, those who had no past antenatal care follow up were more likely to have ANC dropout (AOR = 7.89; 95 % CI: 2.109–29.498) and those who had no professional advice were more likely to have antenatal care dropout (AOR = 4.64 95 % CI: 1.246–17.254).

**Conclusions:**

This study indicates that a high number of women had antenatal care dropout. Having no past ANC follow-up and professional advice were the major factors of ANC service utilization dropout. Hence, giving more information during the ANC visit is important to reduce the dropout rate from the maternity continuum of care.

**Supplementary Information:**

The online version contains supplementary material available at 10.1186/s12884-021-04107-7.

## Introduction

Antenatal care (ANC) is a maternal healthcare service received by pregnant women and adolescent girls from coordinated health care providers to support and sustain the mother’s optimal health during pregnancy, delivery, and puerperium with having and rearing of healthy baby [1]. It has been repeatedly shown to reduce maternal and neonatal deaths via identification of high-risk pregnancies [2–7]. Besides, it provides an opportunity for counseling on nutrition, birth readiness, delivery care, and contraception after birth. It is important particularly in settings and subgroups where the socioeconomic and public health resources are constrained [8, 9].

The World Health Organization (WHO) describes a world in which “every pregnant woman and newborn obtains high-quality care during gestation, labor, and the postnatal period” [10]. In 2015, however, almost 303,000 women and adolescent girls died as a result of pregnancy and delivery difficulties [11]. The majority of maternal deaths occur in low-resource settings, and the majority can be avoided [12]. The WHO recommended at least four ANC visits for pregnant women in 2002 [13]; however this recommendation was changed to at least eight visits in 2016, with the first ANC visit occurring before the 12th week of pregnancy [1].

In Africa, more than two-thirds of pregnant women had at least one prenatal care visit with a health care practitioner [14]. As per the 2019 EMDHS, 74 % of women giving birth in the five years before to the survey had prenatal care from a skilled practitioner at least once during their previous pregnancy. For their most recent live birth, four out of ten women (43 %) had four or more ANC visits. Women in the urban region were more likely than those in the rural area to have had ANC from a skilled provider (85 and 70 %, respectively) and to have had four or more ANC visits (59 and 37 %, respectively) [15]. In Ethiopia and Dire Dawa, the proportion of ANC dropout was 44.58 and 27.4 %, respectively [16]. Over a 14-year period, the percentage of women receiving prenatal care from a skilled provider has increased by 46 % points, from 28 % to 2005 to 74 % in 2019 [15]. While there has been significant progress in Ethiopia in terms of at least one ANC attendance [17, 18], attendance of the recommended visits has been suboptimal [19, 20].

A study conducted on ANC utilization during Corona virus disease 2019 (COVID-19) revealed that only 29.3 % of pregnant women had fully utilized antenatal care services during the pandemic period. Mother age ≥ 35 years secondary education and above history of stillbirth before recent pregnancy, interruption and diversion of services due to COVID-19 response, fear of COVID-19, and lack of transport access were predictors of full antenatal care service utilization [21].

A different cross-sectional study conducted in Ethiopia shows that the key factors of four or more antenatal care services utilization were receiving a high quality of ANC care, having high income, living with a short distance from the health facility, women involving in the household decision making, having a small family size, having good knowledge concerning ANC service, and positive attitude towards ANC services [21–26]. Some studies also showed that age, educational status, occupation; planned recent pregnancy, previous ANC visit, and autonomy on healthcare decision making were found to be significantly associated with ANC utilization [27–29].

It’s known that adequate utilization of the recommended antenatal care visits is important to improve both the health of the mother and the unborn baby. However, there is limited finding on why women fail to use the recommended subsequent ANC visits in Ethiopia especially in the study area. As a result, a context-specific study is important since the country have diversified population based on (ethnicity, culture, belief and religious). Hence, identifying the factors associated with ANC service utilization dropout would have meaningful for intervention. Therefore, this study aimed to assess ANC dropout and associated factors among mothers delivering in public health facilities of Dire Dawa city town.

## Methods and materials

### Study Area

The study was conducted at Dire Dawa; it is one of the Federal city administrations in Ethiopia which is located in the eastern part of Ethiopia, 515 Km from Addis Ababa, the capital city of Ethiopia, and 333 Km from the international port of Djibouti. According to the 2019 population projection, Dire Dawa Administration has 493,000 total populations with 49 % males and 51 % females, out of which around 15,346 were women in the reproductive age group. 68.23 % of the population was urban residents. Dire Dawa administration has 2 Hospitals and 8 Urban Health Centers [30]. All the health centers provide ANC service. There is an average of 4 nurses and 3 midwives that provides ANC services in each health centers.

### Study Design and study period

A facility-based cross-sectional study was employed among mothers who gave delivery in public health facilities of Dire Dawa town from January 1–30, 2021. Therefore, mother who gave delivery at the public health facilities of Dire Dawa town were included in the study. On the other hand mothers who were critically ill and those who were not volunteer to participate were excluded from the study.

### Study Population

All mothers who gave birth at the public health facilities of Dire Dawa town.

### Sample Size Determination

The sample size was calculated using single population proportion formula: n=(Zα/2)^2 *^ p (1-p)/d^2^, considering the following assumptions: prevalence rate of ANC service utilization dropout was taken 44.3 % from EDHS 2014, 95 % confidence interval, 5 % margin of error and correction formula. Hence, the total sample size became 230.

### Sampling procedure

**Fig. 1:**
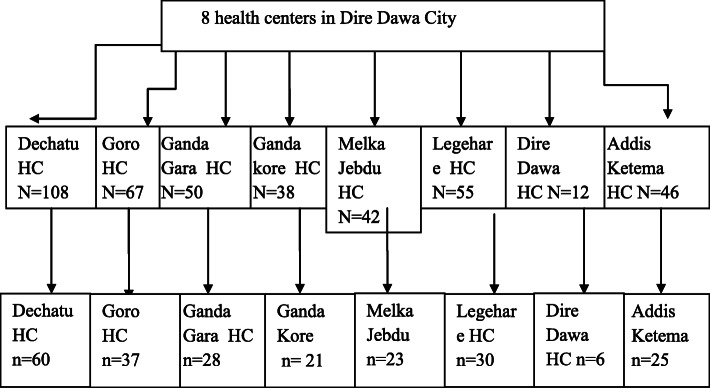
Proportionate fraction of sample size of each health centers of Dire Dawa Town, 2020 (*n* = 230)

Among government health facilities in Dire Dawa town, all health centers in the town were included in the study. The sample size for all health centers was determined according to the principle of proportionate probability technique. The study was conducted in each selected health center to find and register those women who are eligible according to inclusion criteria. Thus, the total participants were identified and the sampled women from each sampling frame were selected using a simple random sampling and the first respondent selected by lottery method while the remaining participants of the study were selected using a systematic sampling technique and a questionnaire was administered to a total of 230 identified delivering mother (Fig. 1).

### Data collection tools and procedures

A pre-tested and structured interviewer-administered questionnaire was used for data collection. Standard questionnaires from EDHS and other similar research done were used [17, 22, 27]. According to the sample allocated to each health center, data was collected during delivery in the selected public health facilities of Dire Dawa town after the pre-test was done in Dil Chora referral hospital. Two Midwives were assigned to collect the data and two BSc degree nurses were supervising data collectors in the process of data collections. However, the sole responsibility of facilitating the whole process was carried out by the principal researcher. The quality of data was assured by using a standard questionnaire from EDHS and other similar research done then translated into Amharic, Afan Oromo, and Somali language. Training was given to the data collectors and supervisors for 2 days on techniques of data collection, data collection material, and purpose of research. Supervision was carried out daily to check data completeness and consistency by both the supervisor and the principal investigators to assure the quality of data.

### Data Entry and Analyses

Data were cleaned, coded, and entered into Epi-info version 7.1 then exported to SPSS version 20 for analysis. A descriptive analysis was carried out to see the distribution of independent variables. A binary logistic regression model with a 95 % confidence interval was used to analyze the results. Bivariable analysis (COR) and multivariable analysis (AOR) was used to analyze the results. From the bivariable analysis, variables with a *p-*value *<* 0.25 were entered into the multivariable logistic regression analysis. From the multivariable logistic regression analysis, variables with a significance level of *p*-value *<* 0.05 were taken as factors independently associated with ANC dropout. Model fitness was checked using Hosmer and Lemeshow goodness of fittest, and a p-value ≤ 0.05 with 95 % confidence interval for Adjusted odds ratio (AOR) was used to determine factors associated with dropout from ANC services.

### Operational Definition

#### ANC dropout

Mothers who did not complete full recommended visit during their pregnancy (a minimum of 4 visits for normal pregnancy) [14].

#### Antenatal care (ANC)

ANC is the care given to a pregnant woman with the aim of improving the maternal and prenatal outcome. ANC includes measuring blood pressure and weights of a woman and taking physical examination, measuring of uterus height and vaccination [31].

#### Quality of service

Respondents were asked whether they had been advised of complications or received certain screening tests during at least one of their antenatal visits [32].

#### Satisfaction

Self reported level of satisfaction stated as satisfied or dissatisfied on exist interviewed of the ANC service received from health professionals [32].

## Results

### Socio demographic characteristics

A total of 230 mothers participated in this study with a 100 % response rate. Eighty-nine (38.7 %) of them were in the age group of 25–29. More than one-third 81 (35.2 %) of the respondent’s husband’s educational status was completed secondary school. Out of the study participants, 79 (34.3 %) had completed primary school followed by secondary school 69 (30 %). The majority of the respondents were Muslim 136(59.1 %) followed by Orthodox 77 (33.5 %). Regarding their ethnicity and occupation, the majority of them were Oromo 129 (56.1 %) and housewives 167 (72.6 %), respectively (Table 1).

**Table 1:** Socio-demographic Characteristics of the respondents in Dire Dawa town, Ethiopia 2020 (*n* = 230)

	Characteristics	Frequency	Percentage
Age	15–19	16	7.0
	20–24	88	38.3
	25–29	89	38.7
	30–34	14	6.1
	35–39	23	10.0
Husband educational status	No formal Education	26	11.3
	Primary	53	23.0
	Secondary	81	35.2
	Diploma and above	70	30.4
Pregnant women educational status	No formal Education	52	22.6
	Primary	79	34.3
	Secondary	69	30.0
	Diploma and above	30	13.0
Religion	Orthodox	77	33.5
	Muslim	136	59.1
	Protestant	17	7.4
Ethnicity	Oromo	129	56.1
	Amhara	46	20.0
	Tigrie	8	3.4
	Guragie	31	13.5
	Somali	16	7.0
Occupation	House wife	167	72.6
	Civil servant	29	12.6
	Merchant	21	9.1
	Student	11	4.8
	Other	2	0.9

### Obstetric condition

More than half 151(65.7 %) of the pregnant mothers were multigravida. Out of this, 79 (52.3 %) had past ANC follow up. Among mothers who had attended for ANC, 56 (70.9 %) of them received ANC service from the health center. Out of the total respondents who had past ANC follow up, the majority 69 (87.3 %) of them delivered at the health centers. Among the participants who attended ANC for the current pregnancy, 144 (62.6 %) of them were started their ANC visit in the first trimester followed by the second trimester 81 (35.2 %). The prevalence of ANC dropout was 86 (37.4 %) (Table 2).

**Table 2:** Obstetric condition of the respondents in Dire Dawa town, Ethiopia 2020 (*n* = 230)

		Obstetric condition	Frequency	Percentage
Parity		Multigravida	151	65.7
		Primigravida	79	34.3
Multigravida	Past ANC follow up	Yes	79	52.3
		No	72	47.7
	Place of past ANC received	Health center	56	70.9
		Hospital	20	25.3
		Private clinic	3	3.8
	Place of delivery for past pregnancy	Health center	69	87.3
		Hospital	8	10.1
		Home	2	2.5
		Private clinic	0	0
	Outcome	Good	78	98.7
		Bad	1	1.3
Time of ANC visit for current pregnancy		First trimester	144	62.6
		Second trimester	81	35.2
		Third trimester	5	2.2
ANC dropout		≤ 3 visit	86	37.4
		≥ 4 visit	144	62.6

### Quality of ANC Services

Concerning the quality of service, 121(52.6 %) of them had satisfied with the service they had received. Out of the respondents, 159 (69.1 %) of them prefer the health center because of its accessibility to transportation. The majority of the respondent 196 (85.2 %) got advice from professionals (Table 3).

**Table 3:** Quality of ANC service in the health center of Dire Dawa town, Ethiopia, 2020. (*n* = 230)

Quality of ANC service		Frequency	Percent
Presence of Professional advice during ANC visit	Present	196	85.2
	Absent	34	14.8
Reason for health center preference	Accessible	159	69.1
	Affordable	15	6.5
	Quality of service	56	24.3
Level of satisfaction	Satisfied	121	52.6
	Dissatisfied	109	47.4

### Associated factors of ANC dropout

Binary logistic regression analysis was used to identify associated factors with ANC service utilization dropout. Past ANC follow-up, a number of family size, planned/supported pregnancy, and professional advice during follow-up were associated in the bivariate analysis but only three (age, past ANC follow-up and professional advice during follow-up) of them were associated in multivariate analysis. Based on the analysis, an age range of 30–39 were less likely to dropout ANC services than those in the age range between 15 and 29 years (*p* = 0.009, at 95 % CI; AOR, 0. 387 [0.189, 0.791]). Concerning past ANC condition, the odds of ANC drop out among women who had no past ANC follow up was 7.89 [2.109–29.498] at 95 % CI. Moreover, those who have no professional advice was more likely to have ANC dropout than those who had professional advice (*p* = 0.022, at 95 %CI: AOR, 4.64[1.246–17.254]) (Table 4).

**Table 4:** Association of selected characteristics with number of ANC visits during gestation, Dire Dawa town, Ethiopia 2020 (*n* = 230)

Characteristics	Number of ANC visits during gestation			COR (95 % CI)	AOR (95 % CI)	p-value
		≥ 4	≤ 3			
Age	15–29	128(88.9 %)	65(75.6 %)	1		
	30–39	16(11.1 %)	21(24.4 %)	0.387[0.189–0.791]*	0.387[0.189–0.791]*	0.009
Number of family size	Two	50(63.3 %)	29(36.7 %)	1		
	≥ 3	94(62.3 %)	57(37.7 %)	0.219[0.115–0.418]*	0.726[0.282–1.865]	0.24
Past ANC history	Had ANC	91(60.3 %)	60(39.7 %)	1		
	No ANC	53 (67.1 %)	26(32.9 %)	0.170[0.063–0.455] *	7.89[2.109–29.498]**	0.002
Current pregnancy planned/supported	Planned	142(64 %)	80(36 %)	1		
	Unplanned	2(25 %)	6(75 %)	0.188[0.037–0.957]*	0.326[0.052–2.060]	0.31
Presence of Professional advice during ANC visit	Present	134(67.3 %)	64(32.7 %)	1		
	Absent	12(35.3 %)	22(64.7 %)	0.264[0.123–0.568]*	4.64[1.246–17.254] **	0.032

NOTE= *; *p*-value ≤ 0.25, and **; *p* –value ≤ 0.05 significant at 95 % CI

## Discussions

This study was aimed at assessing ANC utilization dropout and associated factors in the public health facility, Dire Dawa town Ethiopia. The finding of this research showed that the prevalence of ANC dropout was 37.4 %. This finding was supported by the survey conducted in Dire Dawa, Ethiopia, where ANC drops out was 44.3 % [33]. However, the current study contradicts a study conducted in Nepal, India, where ANC dropout was 50 % to mean less than the recommended number of ANC services for their recent child pregnancy [34]. This discrepancy might be due to the fact that in Ethiopia the role of Health extension workers is crucial by increasing the level of awareness about ANC for pregnant women by their day to day activities or home to home health education.

In the current study, mothers who had no past ANC follow up was significantly associated with current ANC service utilization dropout (AOR = 7.89; 95 % CI: 2.109–29.498). This finding was in agreement with the study conducted in Womberma Woreda, Northwest Ethiopia (AOR = 1.92; 95 % CI: 1.16–3.18) [35]. This might be due to the fact that having past experience of ANC follow-up will encourage a pregnant mother to have ANC visits.

On the other hand, the other factor significantly influences utilization of ANC was the absence of professional advice during ANC visit (64.7 %), which was the main cause to increase ANC dropout rate. This finding was comparable with the study carried out in Addis Ababa and Bahir Dar, where no professional advice during ANC visit with the prevalence of 72.4 and 59.9 %, respectively [36]. In addition, there was significant association of lack of professional advice with ANC dropout (AOR = 0.216: 95 % CI: 0.058–0.803). This finding was supported by other study conducted in Addis Ababa, where there was significant association (AOR = 64; 95 % CI: 1.246–17.254) [37]. Another study conducted in Bahir-Dar special zone revealed that having no professional advice had a significant association to ANC dropout (AOR = 5.47; 95 % CI: 3.10–9.65) [36]. This might be due to the fact that mothers who had professional advice had a clear understanding about antenatal care, their health condition, and their fetal condition as well that make them complete their ANC follow-up.

### Limitation of the study

The current study was limited to small number of study participants and there was also budget limitation to conduct the study with large sample size. Since the study was conducted in the health centers and COVID-19 era, the result may not be applicable to the whole population of delivering mothers in Ethiopia.

## Conclusions

This study revealed that there is a high number of pregnant women had ANC dropout in the study area. Having no past ANC follow-up and absence of professional advice during ANC visit were the major factors of ANC dropout. Hence, giving more information during the ANC visit is important to reduce the dropout rate from the maternity continuum of care.

## Supplementary Information

**Additional file 1:**

## Data Availability

The datasets used and/or analyzed during the current study are available from the corresponding author on reasonable request.

## Abbreviations

ANC: Antenatal care
CSA: Central statistical agency
EDHS: Ethiopian demographic health survey
WHO: World health organization
