# Supplementary material for: Antenatal care dropout and associated factors among mothers delivering in public health facilities of Dire Dawa Town, Eastern Ethiopia
Source: BMC Pregnancy Childbirth. 2021 Sep 15;21:623. doi: 10.1186/s12884-021-04107-7 (PMC8442648; doi:10.1186/s12884-021-04107-7)
Supplement: Supplementary file 1 — Additional file 1: [file 12884_2021_4107_MOESM1_ESM.pdf]

QUESTIONNAIRE

1.1 Health facility _______________

No	1. Socio demographic status	
1.2.	Age of pregnant women	1. 15-19 5.35-39<br>2.20-24 6.40-44<br>3.25-29 7.45-49<br>4.30-34
1.3.	Marital status:	1. Married 3. Divorced<br>2. Single 4. Widowed
1.4.	If married what is the highest level of schooling your husband/partner ever attended?	1. Illiterate<br>2. Can read and write<br>3. Primary<br>4. Secondary<br>5. Diploma and above
1.5.	Educational status of pregnant women	1. Illiterate<br>2. Can read and write<br>3. Primary<br>4. Secondary<br>5. Diploma and above
1.6.	Religion:	1. Orthodox 3. Protestant<br>2. Muslim 4. Other
1.7.	Ethnicity	1. Oromo 4. Guragie<br>2. Amhara 5. Somali<br>3. Tigrie 6.Other
1.8.	Wealth index	1 lowest 4 fourth<br>2second 5 highest<br>3middle
1.9.	What is your occupation?	1. House wife 4. Student

		2. Civil servant 5. Farmer<br>3. Merchant 6. Other Specify
1.10.	Total number of family size	1 one 3. Greater than three<br>2. Two
**1.11.**	Do you have children?	1. Yes 2.no

**2. Obstetric condition**		
**2.1.**	If yes question 1.10, does you has ANC follow up for the past pregnancy?	1.yes 2.no
**2.2.**	If yes Where do you received ANC?	1.Gov'thealthcenter 3.Privetclinic<br>2.Gov't hospital 4.Health post
**2.3.**	If yes question no1.1o, Where do you deliver your baby/s?	1.Gov't health center 4.Health post<br>2.Gov't hospital 5.home<br>3.Privet clinic
**2.4.**	What is the outcome?	1.Good 2.bad
**2.5.**	Is the current pregnancy planned/wanted/ supported?	1.yes 2.no
**2.6.**	Do you have ANC for this Pregnancy in other health facility?	1.Yes 2.no
**2.7.**	Why do you prefer this facility?	1.accessable for transportation<br>2.affordeble<br>3.quality of service<br>4.no choice
**2.8.**	Do you know benefit of ANC follow up?	1.yes 2.no
**2.9.**	If yes where do you get information about the benefit?	1. Health extension 2.media<br>3.both 4. Relatives
**2.10**	Why pregnant mothers once start at health center not complete their follow up at that health center?	1.lack of quality of service<br>2. lack of full investigation

		3. lack of professionals<br>4.other
**2.11.**	How many months pregnant when you first received ANC for the pregnancy?	1. 1st trimenster<br>2.-2nd trimenster<br>3.3rd trimenster
**2.12.**	How many ANC visit for the current pregnancy?	1. One<br>2.Two<br>3.three<br>4.four
**2.13.**	Why do you miss ANC follow up?	1. Lack of knowledge about ANC<br>2.Poor quality of the service<br>3.Others specify
	**3.Knowledge and awareness**	
**3.1.**	Do you think that the professional treat them respectfully?	1.yes 2.no
**3.2.**	Do you think that they get advice on (nutrition complications etc)	1. Yes 2.no
**3.3.**	Where do you need to deliver your baby?	1. Home<br>2. Public Health center<br>3. Privet clinic<br>4.Hospital
	**4.Quality of service**	
**4.1.**	Is there any time that you return back to your home without check up?	1. Yes<br>2.no
**4.2.**	How many time do you spent	1. Less than 20 min<br>2. greater than 20
**4.3.**	.Are you satisfied with the service you received?	1.yes 2.no
**4.4.**	Do you gate all investigation that prescribes	1. Yes 2. no
**4.5.**	If yes how much you satisfied?	1. Good 2.poor
